# Design of a Conceptual Bumper Energy Absorber Coupling Pedestrian Safety and Low-Speed Impact Requirements

**DOI:** 10.1155/2018/9293454

**Published:** 2018-01-14

**Authors:** Fuhao Mo, Siqi Zhao, Chuanhui Yu, Zhi Xiao, Shuyong Duan

**Affiliations:** ^1^State Key Laboratory of Advanced Design and Manufacture for Vehicle Body, Hunan University, Changsha, Hunan 410082, China; ^2^Safety Engineering and Virtual Technology Department, SAIC Motor Technical Center, Jiading District, Shanghai 201804, China; ^3^School of Mechanical Engineering, Hebei University of Technology, Beichen District, Tianjin 300401, China

## Abstract

The car front bumper system needs to meet the requirements of both pedestrian safety and low-speed impact which are somewhat contradicting. This study aims to design a new kind of modular self-adaptive energy absorber of the front bumper system which can balance the two performances. The X-shaped energy-absorbing structure was proposed which can enhance the energy absorption capacity during impact by changing its deformation mode based on the amount of external collision energy. Then, finite element simulations with a realistic vehicle bumper system are performed to demonstrate its crashworthiness in comparison with the traditional foam energy absorber, which presents a significant improvement of the two performances. Furthermore, the structural parameters of the X-shaped energy-absorbing structure including thickness (*t*_u_), side arc radius (*R*), and clamping boost beam thickness (*t*_b_) are analyzed using a full factorial method, and a multiobjective optimization is implemented regarding evaluation indexes of both pedestrian safety and low-speed impact. The optimal parameters are then verified, and the feasibility of the optimal results is confirmed. In conclusion, the new X-shaped energy absorber can meet both pedestrian safety and low-speed impact requirements well by altering the main deformation modes according to different impact energy levels.

## 1. Introduction

The front car bumper system is a complex energy-absorbing system in a car design [1] which must meet both the requirements of pedestrian safety [2, 3] and low-speed impact [4]. An energy absorber is often set between the bumper beam and the bumper skin to absorb impact energy [5–7]. However, the bumper system design requirements of pedestrian safety and low-speed impact are somewhat contradicting regarding force and impact energy levels. Taking the foam bumper energy absorber as an example, the absorber satisfying the low-speed impact well can be generally too stiff when considering the impact with pedestrian lower extremities due to the high force level. On the contrary, the situation is similar. Besides, the traditional energy absorbers are usually an integrated structure made of thermoplastic polymer or foamed polypropylene (EPP) which could need an overall replacement due to a local damage.

In previous studies, several attempts considering pedestrian safety and low-speed impact have been tried [8]. Yao et al. designed a car-front structure on the purpose of pedestrian safety. The structure includes a mechanical cushion in the car bumper for impact energy absorption and a bounce device of hood cover triggered by outer force, and the bumper performance was verified [9]. Wang et al. analyzed the low-speed impact based on dynamic load strength tests of three typical standards of bumper system [10]. Some new bumper systems were designed using new materials [11–14] or structures [15, 16] to achieve the purpose of improving the crashworthiness under the two collision circumstances. In study of Lv et al., a systematic method had been performed to design and optimize the car front-end structure in order to reduce pedestrian injury risks [17]. Shuler designed a new bumper energy absorber using engineering plastics, which included a body and the upper and lower crushable members which would absorb more energy during impact [18]. Mohapatra designed a tunable energy absorber which consists of a frame and a body including a mount of tunable crush lobes to absorb the energy during pedestrian and low-speed impacts [19]. But they featured a complex structure, difficult to manufacture, and still used an integrated structure. Davoodi et al. made a conceptual design and a simulation verification analysis on the bumper energy absorber with fibre-reinforced epoxy polymer composite material [20]. But the energy absorber was mainly in consideration of pedestrian safety without detailed design description for low-speed impact. Therefore, it is expected to design a bumper energy absorber which can well consider the requirements of both pedestrian safety and low-speed impact with evidently different impact energy levels.

Composite material with resin matrix which performs light-weighted, safe, and flexible performance in design and manufacturing is being more and more widely used in vehicle bumper system [21–25]. The present study aims to design an energy-absorbing structure of the bumper system with composite materials which can adaptively adopt different deformation modes according to the amount of impact energy to benefit both pedestrian and low-speed impact. Multiobjective optimization has also been implemented to optimize the conceptual design of this energy-absorbing structure in a realistic family car model, and its results are compared with the original foam absorbing structure.

## 2. Methods and Materials

### 2.1. Conceptual Design of the X-Shaped Energy-Absorbing Unit

**Figure 1:**
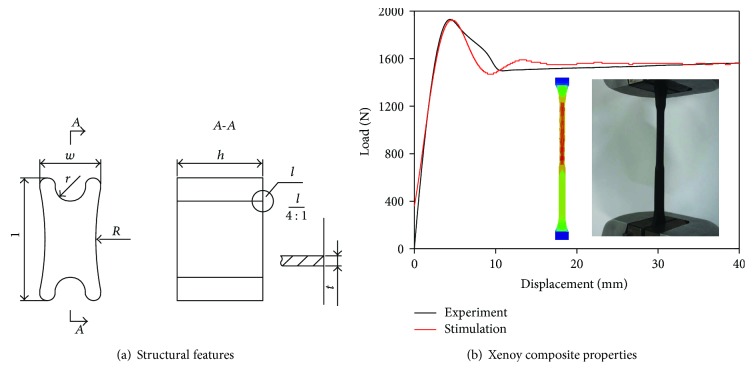
Structural features and material properties of a single X-shaped energy-absorbing unit.

To create a single structure with different energy absorption phases, an X-shaped absorber made of Xenoy composite is proposed as shown in Figure 1. The Xenoy composite (PC/PBT 1103) with a density of 1145 kg/m^3^, elastic modulus of 2317.48 MPa, Poisson's ratio of 0.3, and yield strength of 33.19 MPa is adopted. Its validated simulation parameters of Mat 24 in LS-DYNA codes are presented and validated through the implemented experimental tests using Instron 5984.

**Figure 2:**
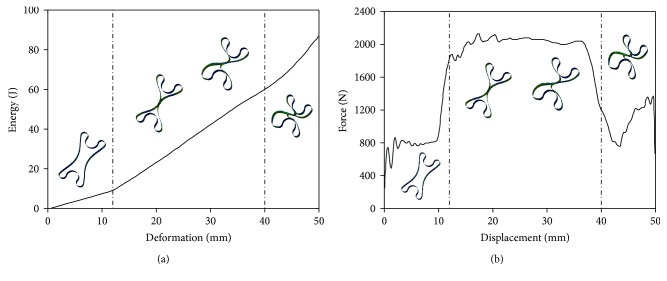
Energy deformation and load deformation curves of X-shaped absorber unit under compression.

Initial geometric parameters of this unit are then determined regarding the vehicle bumper system that would be applied on, with the depth *l* = 80 mm, the width *w* = 40 mm, *R* = 180 mm, *r* = 10 mm, *t* = 2.5 mm, and the height *h* = 56 mm. The compression test is performed on the X-shaped energy absorber with a U shape impactor at a speed of 4 km/h. The compression force and energy-absorbing curves are shown in Figure 2.

During the entire compression process, the X-shaped unit shows different deformation modes with various force levels and energy-absorbing rates. In the deformation stage from 0 to 12 mm, the unit begins to deform to an elastic limit with low force level and low energy-absorbing ability. In 12~40 mm deformation, the two sides of the unit arc get into contact and begin to perform a self-locking status. This leads to a rapid increase of energy-absorbing ability and force levels of the X-shaped unit. In the phase of the deformation higher than 40 mm, the energy absorption unit totally kinks together and is continuously compressed to a deformation limit. Thus, a proper structure design with a number of X-shaped units can be expected to meet different safety requirements under various impact force and energy levels.

### 2.2. Design of Modular Bumper Energy Absorber

**Figure 3:**
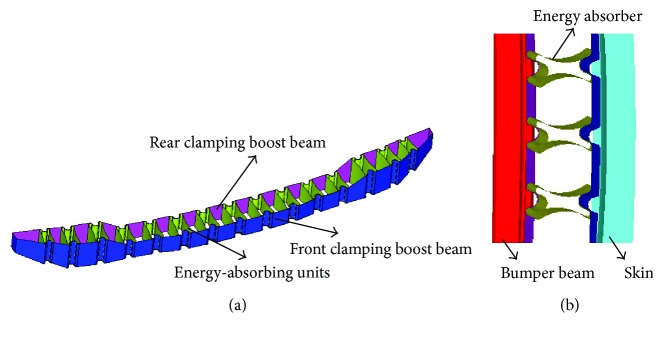
Schematic diagram of the (a) energy absorber and (b) installation position.

With regard to impact energy levels and installation space in the realistic car model, a modular energy absorber is designed as shown in Figure 3(a). It includes fifteen X-shaped units and two clamping boost beams to lock the units between them. The absorber is installed between the bumper skin and bumper beam as the location could be seen in Figure 3(b).

**Figure 4:**
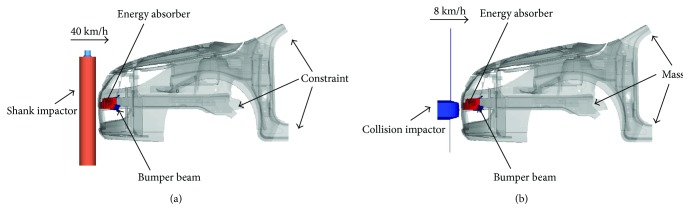
Finite element models of (a) pedestrian lower extremity impact and (b) low-speed impact.

Based on the present car model and energy absorber design, the finite element models of pedestrian lower legform and low-speed impact are established using Hypermesh software as shown in Figure 4 according to the 631/2009/EC regulation [26] and the CMVSS215 regulation, respectively. The impact velocity of the legform is 40 km/h with impact energy at 827.16 J. The low-speed impactor is set at 8 km/h with impact energy at 3207.01 J. Then, impact simulations are initially performed.

### 2.3. Structural Optimization

To further improve the performance of the new bumper system, multiobjective optimization is adopted to determine the structural parameters of the modular energy absorber with X-shaped units. Tests are designed using the full factorial method, input factors are defined as X-shaped unit thickness (*t*_u_), X-shaped unit side arc radius (*R*), and clamping boost beam thickness (*t*_b_) in three levels (Table 1). Output indexes include maximum tibial acceleration (MTA), maximum knee bending angle (MKBA), maximum knee shear displacement (MKSD), collider intrusion (CI), and bumper deformation (BP).

Tests are performed adopting the Hypermesh software, the full factorial experiments are detailedly made then.

## 3. Results and Discussions

**Figure 5:**
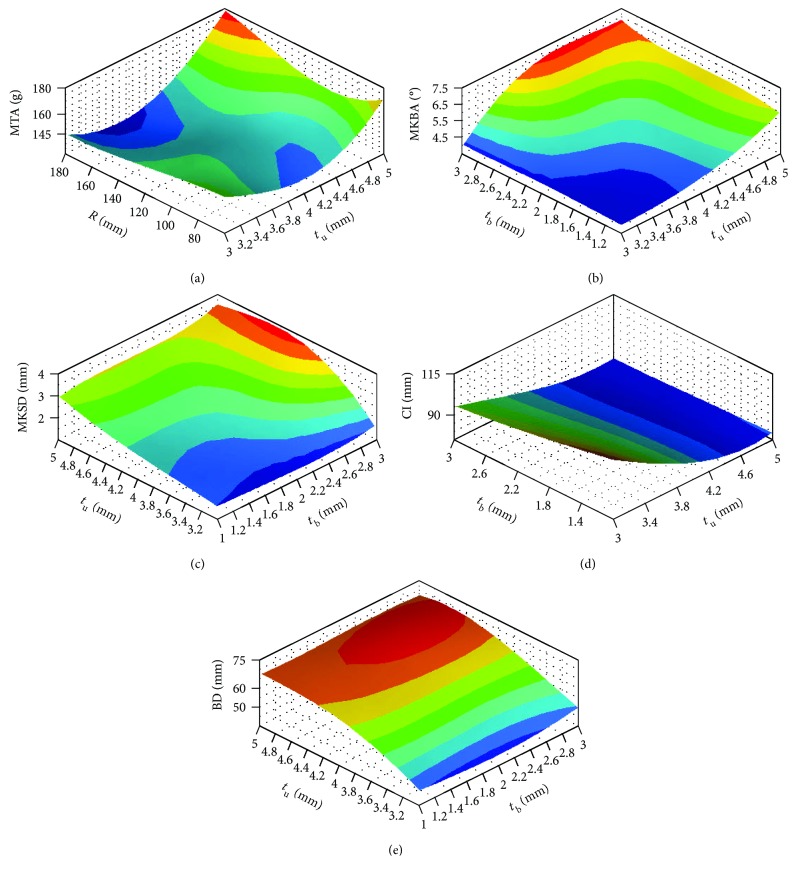
Response surfaces showing simultaneous effects of (a) *t*_u_ and *R* on MTA, (b) *t*_b_ and *t*_u_ on MKBA, (c) *t*_b_ and *t*_u_ on MKSD, (d) *t*_b_ and *t*_u_ on CI, and (e) *t*_b_ and *t*_u_ on BD.

The overall results of low-speed impact and pedestrian safety tests are listed in Table 2. The correlation of output index values to input structural parameters is shown in Figure 5. As can be visualized in Figure 5, *t*_u_ is the most influential parameter of all these factors. MTA is also greatly influenced by *R*, while the effect of *t*_b_ is less. MKBA, MKSD, BD, and CI are affected by *t*_b_ a lot and the influence of *R* is slight.

Regarding pedestrian safety tests, Figure 5(a) reveals the interaction effect between *t*_u_ and *R* on MTA. The MTA value considerably increases with the increase of *t*_u_ at high levels *t*_u_ from approximately 4.2 mm to 5 mm. On the contrary, the decline of *t*_u_ leads to the decrease of the MTA at low *t*_u_ values. The influence of *R* on the MTA is less. For the values of *R* from 80 mm to 180 mm, the MTA increases initially and then decreases. The minimum MTA of 130 g is obtained at approximately 3.8 mm *t*_u_ and 180 mm *R*. The changes of the MKBA value on *t*_b_ and *t*_u_ are presented in Figure 5(b). It presents that increasing *t*_u_ leads to decrease of the MKBA. Similarly, the MKBA slightly increases with the decline of *t*_b_. The minimum MKBA of approximately 4° is obtained at 3 mm *t*_u_ and 2 mm *t*_b_. The dependence of MKSD on *t*_b_ and *t*_u_ is presented in Figure 5(c). It is observed that the MKSD notably increases with the increase in *t*_u_ and is slightly influenced by *t*_b_.

For low-speed impact tests, Figure 5(d) plots the influences of *t*_b_ and *t*_u_ on CI. The CI decreases from 95 mm to 78 mm with the increase of *t*_u_ from 2 mm to 5 mm while the effects of *t*_b_ on CI are less. The effect of *t*_u_ and *t*_b_ on BD values can be visualized in Figure 5(e). It is revealed that BD increases to a maximum point and then decreases with *t*_u_ from 3 mm to 5 mm. BD has a gentle increase with the increase of *t*_b_. The maximum BD of approximately 70 mm is obtained at 4.8 mm *t*_u_.

After this, we adopt a set of samples to ensure that the accuracy of the Kriging model is accepted. We use four criteria to judge the accuracy of the model: *R*-squared (*R* ^2^), root mean square error (RMSE), relative average absolute error (RAAE), and relative maximum absolute error (RMAE). The values are 0.999, 0.131, 0.492, and 0.009, respectively. It can be observed that this model is relatively accurate and can be used for the subsequent optimization model.

Then, the multiobjective particle swarm optimization algorithm including 511 iterations is selected to optimize the design variables. Then, a relatively good result was selected among the results, and the optimization results are shown in Table 3. Since the above results are based on the optimization results of the algorithm, analyses are performed to verify the obtained structural parameters. The three optimal structural parameters are substituted to the original finite element model of pedestrian safety and low-speed impact. Two contrast simulation models are established and the evaluation results are shown in Table 3.

As shown in Table 3, all damage index values of the optimized structure are superior to the initial solution while satisfying the requirements of the regulations. The error of the value between the final verification and the optimal solution is controlled within 15%. This indicates that the optimization method used in this study is reliable.

**Figure 6:**
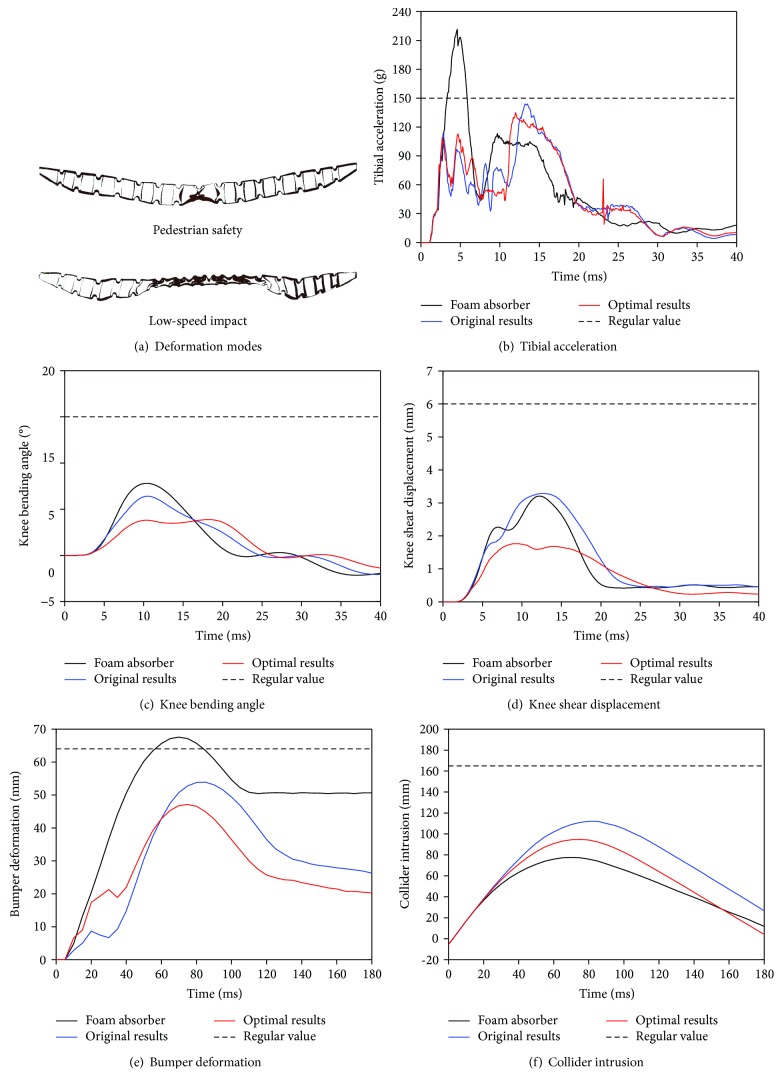
Comparison of evaluation index values regarding pedestrian safety and low-speed impact.

The performances of pedestrian safety and low-speed impact protection based on the traditional foam absorber, the original X-shaped energy absorber model, and the optimal verification model are compared and shown in Figure 6. It should be noted that most risk index values of the impact simulations with X-shaped energy absorbers are reduced including all below the corresponding thresholds compared to those of the impact simulations with the traditional foam absorber. One of the most important reasons can be due to dual deformation modes of the X-shaped energy-absorbing unit during various impacts with different amounts of energy. In the pedestrian safety test, the units absorb energy mainly before forming the self-locking structure and effectively decline the peak value of the impact force. In the low-speed impact test, the X-shaped units absorb energy mainly by the self-locking mode with higher energy-absorbing efficiency.

It can be observed in Figure 6(b) that at 4 ms, the leg impactor gets into contact with the bumper skin which leads to an elastic deformation of the X-shaped energy absorber; the first peak is obtained. At about 7 ms, the X-shaped energy absorber reaches the elastic limits after compressing and forms the second peaks. Further, when the X-shaped energy absorption unit exceeds the elastic limit to 13 ms, the two arc sides get into contact with each other to form a third peak. At 40 ms, the energy of the X-shaped energy absorption unit is gradually released, resulting in a certain rebound.

Figure 6(b) shows that the X-shaped energy absorber shows an evidently better energy absorption performance when compared with the foam absorber. After using the new energy absorber with the X-shaped units, the maximum tibial acceleration related to pedestrian protection decreases notably to 127 g. As shown in Figure 6(a), the impact load is distributed to different compression stages to achieve the purpose of reducing damage with multiple peaks instead of a large acceleration peak of the traditional foam energy absorber. When the leg impactor comes into contact with the bumper skin and the X-shaped energy absorption unit begins to compress, the tibial acceleration curve obtains the first peak. Then, the energy absorber is continuously compressed until its elasticity limit and until the second acceleration peak is formed. Further, the elastic limit is exceeded and a self-locking status of the X-shaped unit is formed; the third peak is obtained. The maximum knee bending angle and shear displacement are also significantly reduced by 50% (Figures 6(c) and 6(d)) to 4.5° and 1.93 mm, respectively. All these indicate that the X-shaped bumper energy absorber adaptively adopts the small deformation mode in the pedestrian safety test due to the low impact energy.

In the low-speed impact test as shown in Figure 6(e), the maximum deformation of the bumper has a significant decline when comparing the new X-shaped energy absorber with the traditional foam absorber. At the initial stages of 0~30 ms, the X-shaped units are in the deformation phase before two arcs are in contact and the two sides of the arc are in contact with each other to form a self-locking structure, reaching a peak of 90 ms while the energy absorption capacity rapidly increases. It is revealed that the new bumper energy absorber adaptively adopts the large deformation mode in the low-speed collision test, which absorbs more energy and significantly reduces the bumper deformation peak, as shown in Figure 6(a). In Figure 6(f), the maximum value of the collider intrusion has also been largely reduced due to the structure optimization.

All of the above indicates that the new X-shaped energy absorber shows a better performance in the present bumper system compared to the traditional foam absorber, in particular to provide an effective force and energy-absorbing control through different deformation modes. Meanwhile, due to the modular design, only the damaged bumper energy-absorbing units during the impact need to be replaced and the other units remaining intact can be used again which means that the new energy absorbers are easy to repair in an economical way.

In addition, the parameters of pedestrian safety and low-speed impact are greatly improved after applying the structural parameters obtained by the optimization algorithm in this study. For pedestrian safety, the maximum MTA decreases from 143.6 mm to 134.5 mm, the maximum MKBA decreases from 6.41° to 3.88° with a reduction of 39.47%, and the maximum MKSD decreased from 3.29 mm to 1.76 mm with a reduction of 46.50%. For low-speed impact, the maximum CI decreases from 111.93 mm to 94.81 mm with a reduction of 15.30%. The maximum value of BD reduces from 53.91 mm to 47.09 mm with a reduction of 12.65%. All these indicates the efficiency and contributions of the multiobjective optimization method used in the design of the new energy absorber with the X-shaped unit.

## 4. Conclusions

This paper proposes and designs a new conceptual type of bumper energy absorber in a multioptimization method considering the requirements of both pedestrian safety and low-speed impact, which adopts a modular design in the form of assembling with an X-shaped unit. This unit type presents grading deformation modes with different energy-absorbing rates and force levels. The results reveals that the new bumper energy absorber proposed in this paper adaptively uses different energy absorption modes in different collision forms based on the structural characteristics of its own X-shaped unit and rapidly increases the energy absorption capacity after self-locking. So, it performs a better comprehensive performance compared to the traditional foam-type energy absorber by effectively controlling the force level and energy-absorbing rate. The modular design also indicates its easy changing and fixing.

Besides, the multiobjective optimization of the structural parameters is performed for the detailed design of the new bumper energy absorber. The pedestrian protection and low-speed impact performance of the new energy absorber with optimized structural parameters are greatly improved, and the requirements of pedestrian safety and low-speed impact are better balanced.

## Figures and Tables

**Table 1:** Levels of structural parameters.

Number	Factor	Case1	Case2	Case3
A	*t*_u_	3 mm	4 mm	5 mm
B	*R*	60 mm	120 mm	180 mm
C	*t*_b_	1 mm	2 mm	3 mm

**Table 2:** Design of experiments with experimental conditions.

Run	A	B	C	MTA (g)	MKBA (°)	MKSD (mm)	CI (mm)	BD (mm)
1	1	1	1	139.1	4.46	2.40	113.47	49.70
2	1	2	2	149.3	4.05	1.40	102.02	44.72
3	1	3	3	124.3	4.11	1.60	101.35	44.22
4	2	1	2	138.5	4.71	2.09	85.69	64.79
5	2	2	3	148.3	7.11	3.97	82.08	65.26
6	2	3	1	140.9	4.56	2.50	83.44	54.66
7	3	1	3	171.5	6.86	3.24	80.56	72.83
8	3	2	1	162.8	6.02	2.97	79.04	67.56
9	3	3	2	179.0	6.42	3.27	78.76	68.43
10	1	1	2	156.4	4.01	1.48	106.99	47.42
11	1	1	3	130.8	4.14	1.66	101.77	50.24
12	1	2	1	127.3	3.93	1.54	110.68	46.73
13	1	2	3	124.9	3.99	1.59	95.75	49.35
14	1	3	1	148.6	4.42	1.61	110.17	46.62
15	1	3	2	143.6	4.00	1.44	101.55	44.37
16	2	1	1	138.1	4.87	2.33	90.84	42.60
17	2	1	3	143.4	5.28	2.38	84.15	67.08
18	2	2	1	133.5	4.33	2.06	84.34	64.84
19	2	2	2	144.6	4.64	2.14	83.30	65.90
20	2	3	2	135.9	4.55	2.13	82.43	64.30
21	2	3	3	141.7	5.15	2.39	80.90	64.01
22	3	1	1	164.3	6.06	2.86	87.40	68.89
23	3	1	2	170.9	6.32	2.93	81.03	69.03
24	3	2	2	159.4	6.39	3.14	78.10	68.31
25	3	2	3	161.2	6.89	3.58	77.35	68.04
26	3	3	1	183.8	6.02	2.96	79.59	68.43
27	3	3	3	194.9	6.89	3.68	77.90	68.70

**Table 3:** Multiobjective optimization results and verification.

Variables	A	B	C	MTA (g)	MKBA (°)	MKSD (mm)	CI (mm)	BD (mm)
Regular value	—	—	—	150.0	15.00	6.00	165.00	64.00
Foam absorber	—	—	—	221.58	7.80	3.21	77.78	67.56
Original results	2.5	180.0	2	143.6	6.41	3.29	111.93	53.91
Optimal results	3.2	146.4	3	127.0	4.50	1.93	93.55	51.55
Verification	3.2	146.4	3	134.5	3.88	1.76	94.81	47.09
Deviation	—	—	—	5.91%	13.78%	8.81%	1.35%	8.65%

## References

[1] Davoodi M. M., Sapuan S. M., Aidy A., Abu Osman N. A., Oshkour A. A., Wan Abas W. A. B. (2012). Development process of new bumper beam for passenger car: a review. *Materials & Design*.

[2] Gil H. M., Kwon Y. D., Kim D. H., Kim Y. S. (2016). Minimizing pedestrian lower-leg injury considering rate dependence of the plastic energy absorber. *International Journal of Automotive Technology*.

[3] Abvabi A., Nasr A., Noorpoor A., Kiasat M. S. (2010). Lower extremity injuries in vehicle-pedestrian collisions using a legform impactor model. *Journal of Zhejiang University Science A*.

[4] Wang B., Yang J., Otte D. The effects of vehicle front design variables and impact speed on lower extremity injury in pedestrian collisions using in-depth accident data.

[5] Zhang Q., Zhao L., Zhang Q., Wei X. Geometry parameter optimization method for automobile energy-absorbing box.

[6] Wang Y., Wang L., Ma Z., Wang T. (2016). A negative Poisson’s ratio suspension jounce bumper. *Materials & Design*.

[7] Xiao Z., Fang J., Sun G., Li Q. (2015). Crashworthiness design for functionally graded foam-filled bumper beam. *Advances in Engineering Software*.

[8] Zeng F., Xie H., Liu Q., Li F., Tan W. (2016). Design and optimization of a new composite bumper beam in high-speed frontal crashes. *Structural and Multidisciplinary Optimization*.

[9] Yao J., Liu M., Lu W., Luan J. Optimization design of car front structure based on pedestrian protection.

[10] Wang Z., Chen L. Dynamic and energy analysis based on bumper system low-speed collision test.

[11] Belingardi G., Beyene A. T., Koricho E. G., Martorana B. (2015). Alternative lightweight materials and component manufacturing technologies for vehicle frontal bumper beam. *Composite Structures*.

[12] Davoodi M. M., Sapuan S. M., Ahmad D., Aidy A., Khalina A., Jonoobi M. (2011). Concept selection of car bumper beam with developed hybrid bio-composite material. *Materials & Design*.

[13] Belingardi G., Beyene A. T., Koricho E. G. (2014). Geometrical optimization of bumper beam profile made of pultruded composite by numerical simulation. *Composite Structures*.

[14] Montoya M. C., Costas M., Díaz J., Romera L. E., Hernández S. (2015). A multi-objective reliability-based optimization of the crashworthiness of a metallic-GFRP impact absorber using hybrid approximations. *Structural and Multidisciplinary Optimization*.

[15] Shin M. K., Yi S. I., Kwon O. T., Park G. J. (2008). Structural optimization of the automobile frontal structure for pedestrian protection and the low-speed impact test. *Proceedings of the Institution of Mechanical Engineers, Part D: Journal of Automobile Engineering*.

[16] Park D. K., Jang C. D., Lee S. B., Heo S. J., Yim H. J., Kim M. S. (2010). Optimizing the shape of a bumper beam section considering pedestrian protection. *International Journal of Automotive Technology*.

[17] Lv X., Gu X., He L., Zhou D., Liu W. (2015). Reliability design optimization of vehicle front-end structure for pedestrian lower extremity protection under multiple impact cases. *Thin-Walled Structures*.

[18] Shuler S. F., Surisetty G. K., Nanda A. (2007). *U.S. Patent No. 7,163,242*.

[19] Mohapatra S., Nanda A., Mooijman F. (2007). *U.S. Patent No. 7,278,667*.

[20] Davoodi M. M., Sapuan S. M., Yunus R. (2008). Conceptual design of a polymer composite automotive bumper energy absorber. *Materials & Design*.

[21] Liu Z., Lu J., Zhu P. (2016). Lightweight design of automotive composite bumper system using modified particle swarm optimizer. *Composite Structures*.

[22] Szlosarek R., Bombis F., Mühler M., Kröger M., Karall T. (2016). Development of carbon fibre-reinforced plastic (CFRP) crash absorbers with stable crushing behaviour considering the connection to the bumper system. *Materialwissenschaft und Werkstofftechnik*.

[23] Kim D. H., Kim H. G., Kim H. S. (2015). Design optimization and manufacture of hybrid glass/carbon fiber reinforced composite bumper beam for automobile vehicle. *Composite Structures*.

[24] Hosseinzadeh R., Shokrieh M. M., Lessard L. (2006). Damage behavior of fiber reinforced composite plates subjected to drop weight impacts. *Composites Science and Technology*.

[25] Beyene A. T., Koricho E. G., Belingardi G., Martorana B. (2014). Design and manufacturing issues in the development of lightweight solution for a vehicle frontal bumper. *Procedia Engineering*.

[26] The European Parliament and the Council of the European Union (2009). Regulation (EC) no 78/2009 of the European Parliament and of the council of 14 January 2009. *Official Journal of the European Union*.

